# One-Pot Thermal Synthesis of g-C_3_N_4_/ZnO Composites for the Degradation of 5-Fluoruracil Cytostatic Drug under UV-LED Irradiation

**DOI:** 10.3390/nano12030340

**Published:** 2022-01-21

**Authors:** Álvaro Pérez-Molina, Luisa M. Pastrana-Martínez, Lorena T. Pérez-Poyatos, Sergio Morales-Torres, Francisco J. Maldonado-Hódar

**Affiliations:** NanoTech—Nanomaterials and Sustainable Chemical Technologies, Department of Inorganic Chemistry, Faculty of Sciences, University of Granada, Avda. Fuente Nueva, s/n, ES-18071 Granada, Spain; alpemo@ugr.es (Á.P.-M.); lpastrana@ugr.es (L.M.P.-M.); lorenaperez@correo.ugr.es (L.T.P.-P.); fjmaldon@ugr.es (F.J.M.-H.)

**Keywords:** carbon nitride, zinc oxide, photocatalysis, 5-fluorouracil, water treatment, scavengers

## Abstract

Graphitic carbon nitride (g-C_3_N_4_) was used to enhance the photocatalytic activity of ZnO nanoparticles for the degradation of 5-fluorouracil (5-FU) cytostatic drug under UV-LED irradiation. CN/ZnO composites were synthetized by an easy one-pot thermal method, varying the g-C_3_N_4_ loading, i.e., from 10 to 67 wt% and a post-thermal exfoliation in air. The physicochemical and optical properties of the materials were analyzed by several techniques. CN/ZnO composites showed a coral-like structure of spherical ZnO wurtzite particles on the g-C_3_N_4_ structure. In general, the synergism and heterojunction interface between both phases allowed the enhancement of the mesoporosity, light absorption ability, and the aromaticity of the corresponding composites. Moreover, the photocatalytic activity of the CN/ZnO composites was increased with the addition of g-C_3_N_4_ in comparison with pristine ZnO. The highest activity was found for the composite containing 25 wt% of g-C_3_N_4_ (i.e., CN25/ZnO), reaching the total degradation of 5-FU and a mineralization of 48% at 180 min, as well as a good photostability during four reuse cycles. Experiments with different pH solutions and scavengers allowed for the assessment of the reactive oxygen species (ROS) involved in the 5-FU degradation pathway, with radicals and non-radical species as the main responsible active species. Furthermore, a tentative photocatalytic mechanism was proposed for CN/ZnO composites.

## 1. Introduction

An intensive agriculture and livestock activity, as well as increased industrial discharges and a growing population play a prominent role in the decrease of natural water resources quality. This leads to a marked impact in the middle or long-term effect on the environment, wild life, and human health. During the last decades, a vast number of pollutants has been detected in effluents from municipal wastewater treatment plants (WWTPs), river water, seawater, groundwater and even, tap water [1]. Contaminants of emerging concern (CECs) include a wide family of compounds, such as dyes, pesticides, pharmaceuticals, food additives, personal care products, industrial compounds, hormones, endocrine disruptors, and so on. [2]. Cytostatic drugs, also known as cytotoxic, antineoplastic or anticancer drugs, are a group of pharmaceuticals extensively used to prevent the growth or to inhibit cancer cell activity. In general, pharmaceuticals are not completely metabolized by the patients, part of them are excreted through the urine and/or feces. Furthermore, these pollutants cannot be removed efficiently in conventional WWTPs and as a consequence, are discharged and accumulated into seas and oceans. The 5-Fluorouracil (5-FU) is the active substance of capecitabine pharmaceutical and is classified as an antimetabolite due to its inhibitory mechanism of nuclear RNA exosome complex and tumor growth [3,4]. The physicochemical properties of 5-FU are relevant for the determination of its environmental action and the effects on human health and wild life. In fact, this pharmaceutical can be persistent in water resources as a consequence of its high mobility in sediment and/or soil (K_oc_ 8), low adsorption into suspended solids, and small octanol–water partition coefficient (K_ow_ 10^–0.89^) [5]. Therefore, 5-FU is one of the most commonly used drugs in cancer therapy and has been detected in hospital wastewater at low concentrations (µg L^–1^) and natural waters (ng L^–1^) from most of European, North, and South American countries, as well as Japan and China [6,7].

Advanced oxidation processes (AOPs) have proven their effectiveness for the removal of pollutants in water through the generation of highly reactive radicals (e.g., hydroxyl radicals, **^−^**OH). Among AOPs, heterogeneous photocatalysis is an attractive option since it can be applied using sunlight and non-toxic materials, resulting in a carbon footprint reduction. In the last years, graphitic carbon nitride (g-C_3_N_4_) has emerged as an interesting photocatalyst due to its effective light harvesting, accompanied by a narrow band gap (i.e., 2.7 eV) and high photocatalytic activity for the degradation of different organic pollutants in aqueous solution [8]. This polymeric semiconductor presents strong carbon–nitrogen covalent bonds providing a high chemical and thermal stability and high electronic conductivity due to a delocalized conjugated structure [9]. Nevertheless, its practical application is still limited by the low light absorption coefficient, poor porosity, and low-charge carrier mobility. Different strategies have been investigated to improve the chemical and texture properties, as well as the charge separation of g-C_3_N_4_, namely, using different precursors [10], synthesis, and post-treatment techniques [11,12], doping with metals and non-metals [13,14], in combination with carbon materials [15,16,17,18] and semiconductor heterojunction [19,20], among others.

An effective approach for promoting the charge transfer and retarding the electron-hole recombination rate is the development of composites using two different semiconductors with appropriate valence and conduction band potentials. Zinc oxide (ZnO) is a widely investigated photocatalyst with comparable properties to TiO_2_, such as non-toxic nature, high activity under UV radiation, low cost [21,22], and rapid recombination of photogenerated electron–hole pairs [23,24]. Furthermore, ZnO can be hybridized with g-C_3_N_4_ resulting in g-C_3_N_4_/ZnO composites with an enhanced visible radiation absorption and charge separation in the electron transfer process [8,9,25]. Several methods have been studied to synthetize g-C_3_N_4_/ZnO composites for different photocatalytic applications, namely photoreduction of CO_2_, production of H_2_ by water spitting, degradation of organic pollutants, etc. Zhu et al. [26] reported the synthesis of carbon-doped g-C_3_N_4_/ZnO composites by the one-step calcination method using dicyandiamide as precursor of g-C_3_N_4_ and a carbon source to replace the lattice oxygen of the ZnO structure. The best catalytic performance was obtained with a catalyst containing a ~50 wt% of g-C_3_N_4_ for the degradation of methylene blue (MB) under visible radiation (*λ* > 400 nm). The same g-C_3_N_4_ content was also found in the g-C_3_N_4_/ZnO composites for the degradation of methyl orange [27] and amiloride [28] under visible light irradiation. Nevertheless, other authors have reported the best catalytic performance for g-C_3_N_4_/ZnO composites containing a lower g-C_3_N_4_ content [29,30,31,32]. Therefore, Liu et al. [29] concluded that 5.0 wt% of g-C_3_N_4_ in g-C_3_N_4_/ZnO composites led to the highest activity for the photooxidation of rhodamine B (RhB) and the photoreduction of Cr^6+^ under visible light irradiation, while 10 wt% of g-C_3_N_4_ was determined to be the optimum amount for the removal of RhB over g-C_3_N_4_ coated hollow pencil-like ZnO composites [32]. Recently, Naseri et al. [31] prepared g-C_3_N_4_/ZnO nanofibers via an electrospinning technique for the degradation of MB under simulated solar radiation. The best catalytic performance was achieved with a composite containing 0.25 wt% of g-C_3_N_4_. 

Overall, the photocatalytic performance of g-C_3_N_4_/ZnO composites is not only affected by different synthesis conditions, such as the C_3_N_4_ precursor, the employed method, the g-C_3_N_4_/ZnO ratio, but also the type of pollutant and the irradiation source studied. In the present work, the one-pot thermal decomposition route was studied for the synthesis of g-C_3_N_4_/ZnO composites using melamine and zinc nitrate, as precursors of g-C_3_N_4_ and ZnO, respectively. The optimum g-C_3_N_4_/ZnO ratio was studied for the photodegradation of the 5-FU cytostatic drug under UV-LED irradiation. The 5-FU contaminant has not been previously studied with g-C_3_N_4_ materials, while UV-LED systems were demonstrated as energetically efficient, compact, do not heat the solution and with longer half-life and higher quantum yield than the conventional illumination systems based on UV–Vis lamps. The physicochemical, crystallographic and optical properties of the composites were studied by several techniques with a special emphasis on the investigation of possible correlations and synergistic effects between the properties and composition of the composites. Additional photocatalytic experiments using different scavengers and consecutive reaction cycles were carried out to assess the possible reaction mechanism and the photocatalyst stability, respectively. 

## 2. Materials and Methods

### 2.1. Synthesis of g-C_3_N_4_/ZnO Composites

Graphitic-C_3_N_4_/ZnO composites (hereafter CN/ZnO) were prepared by an easy thermal treatment for the simultaneous decomposition of melamine (C_3_H_2_N_6_, 99%, VWR Chemicals, Leuven, Belgium) and zinc nitrate tetrahydrate (Zn(NO_3_)_2_ 4H_2_O, 98.5%, Merck, Darmstadt, Germany) following a methodology adapted from [11]. Melamine was selected as the g-C_3_N_4_ precursor taking into account preliminary results (not shown) and as cheaper than other typical precursors reported in the literature, such as urea, thiourea, cyanamide, etc. In a typical procedure, a certain amount of melamine and Zn(NO_3_)_2_ was mechanically mixed for 10 min and the resulting mixture was placed in a semi-closed crucible inside a muffle furnace (Carbolite, ELF 11/148 model, Hope Valley, UK) under static air atmosphere at 2 °C min^−1^ up to 450 °C for 2 h. Then, the temperature was increased up to 550 °C for 4 h under the same experimental conditions. Finally, the synthetized materials were washed repeatedly with distilled water in order to remove the unreacted precursors, and then dried at 60 °C overnight. 

Subsequently, a thermal post-treatment over the composites was carried out with some portions of the samples in a semi-open crucible placed into a muffle furnace at 500 °C for 2 h under static air atmosphere, with the aim of increasing the surface area and the charge separation efficiency of the resulting composites [11]. The mass ratio of melamine/Zn(NO_3_)_2_ was changed in order to obtain CN/ZnO composites with 10, 25, 50, and 67 wt% of g-C_3_N_4_, the samples were referred as CN10/ZnO, CN25/ZnO, CN50/ZnO, and CN67/ZnO, respectively. For comparison purposes, pure g-C_3_N_4_ (sample CN) and pristine ZnO were also synthetized following the same methodology, but without any amount of Zn(NO_3_)_2_ and melamine, respectively.

### 2.2. Characterization Techniques

The X-ray diffraction (XRD) patterns were recorded by a Philips PW 1710 diffractometer (Bruker, Rivas-Vacia, Madrid, Spain) using the CuK*α* radiation and a nickel filter that removes the κβ radiation. The crystallite size (*d*_ZnO_) of the materials was calculated by applying the Scherrer equation to the highest diffraction peak, which was corresponding to the (101) lattice plane of the hexagonal wurtzite structure (JCPDS card no. 36-1451). Fourier-transform infrared spectra (FTIR) were recorded in a NICOLET 510P spectrometer (Thermo Fisher Scientific, Waltham, MA, USA) with an attenuated total reflection (ATR) accessory and a ZeSn as ATR crystal. The N_2_ adsorption–desorption isotherms at—196 °C were carried out using the Quadrasorb SI equipment (Quantachrome, Boston Beach, FL, USA). Previously, the samples were outgassed under high vacuum (10^−6^ mbar) at 110 °C for 12 h. The Brunauer–Emmett–Teller (BET) equation was applied to calculate the apparent surface area (*S*_BET_) [33,34]. Total pore volume (*V*_total_) and mesopore volume (*V*_meso_) were determined by applying the Barret–Joyner–Halenda (BJH) method [35] to the desorption branch. The morphology of the prepared materials was studied by scanning electron microscopy (SEM) using a LEO (Carl Zeiss) GEMINI-1430VP microscope (Oberkochen, Germany). X-ray photoelectron spectroscopy (XPS) was carried out using a Physical Electronics VersaProbe II apparatus (PHI, Chanhassen, MN, USA) equipped with a MgK*α* X-ray source (*hν* = 1486.6 eV) working at 1.3 V and 20 mA, and a hemispherical electron analyzer. Survey and multi-region spectra were recorded at the C1s, N1s, O1s, and Zn2p photoelectron peaks. The optical properties of photocatalysts were studied by the UV–Vis spectrophotometer CARY 5E (VARIAN, Palo Alto, CA, USA) equipped with a diffuse reflectance accessory (DRA). The spectra in diffuse reflectance mode were transformed to equivalent Kubelka–Munk units. The band gap of the photocatalysts was determined from the corresponding Tauc’s plots using Kubelka–Munk units (K–M·E)^1/2^ as a function of energy (eV) [36]. The point zero of charge (pH_PZC_) of the materials was determined following a pH drift test, as described in [37,38]. Briefly, nitrogen was first bubbled in distillate water with the aim of the prevention of carbon dioxide dissolution and respective water acidification. Then, solutions with varying initial pH (2–12) were prepared using HCl (0.1 mol L^−1^) or NaOH (0.1 mol L^−1^) and 50 mL of NaCl (0.01 mol L^−1^) as electrolyte. Each solution was contacted with 0.10 g of the material and the final pH was measured after 24 h of continuous stirring at room temperature. The PZC value of the material was determined by intercepting the obtained final-pH vs. initial-pH curve with the straight line final-pH = initial-pH.

### 2.3. Photocatalytic Experiments

The photocatalytic performance of the materials was studied for the degradation of 5-FU (C_4_H_3_FN_2_O_2_, >99%, Tokyo Chemical Industry, Tokyo, Japan) in aqueous solution under UV-LED irradiation and at room temperature. The photocatalytic tests were carried out in a refrigerated glass reactor loaded with 100 mL of aqueous solution containing the 5-FU cytostatic drug (154 mM). The photocatalyst load was fixed as 1 g L^–1^ and the suspension was continuously stirred with a magnet and with an oxygen flow as purge. The oxygen was allowed to saturate the photocatalyst suspension in order to obtain a high dissolved oxygen concentration in the reaction media, since it has an influence on the photocatalytic activity [39]. The irradiation was performed using a 10 W high-intensity UV-LED (15.5 × 23 mm) with a maximum irradiance wavelength at 385 nm. In order to achieve the adsorption–desorption equilibrium, the suspension was maintained in a dark period for 30 min before turning on the LED. The reaction in the absence of photocatalysts (photolysis) was also carried out under similar experimental conditions. Moreover, an additional experiment with nitrogen purge rather than oxygen continuous flow was performed for comparison purposes.

During the adsorption and reaction stages, small aliquots were periodically withdrawn from the reactor at different time intervals and the photocatalyst was separated with a 0.45 µm polyethersulfone (PES) filter (Agilent Technologies, Santa Clara, CA, USA). In all of the photocatalytic experiments, the concentration of 5-FU (*λ*_max_ = 265 nm) was monitored by ultra-high performance liquid chromatography (UHPLC), using the Shimadzu Corporation apparatus (Nexera model, Tokyo, Japan) equipped with a pump LC-30AD, an autosampler SIL-30AC, an oven CTO-20AC, a degasser DGU-20A5r, a system controller CBM-20 A Lite, and a diode array detector (SPD-M20A). Chromatographic separation was carried out using a Shim-pack GISS-HP C18, 3 µm column (100 × 3.0 mm ID) provided by Shimadzu Corporation (Tokyo, Japan). The injection volume was 20 µL, while the temperature of the column oven and autosampler was set at 30 and 15 °C, respectively. The mobile phase consisted of a mixture of water and methanol (97:3 *v*/*v*) at isocratic mode and a flow rate of 0.2 mL min^–1^.

The photocatalytic degradation was calculated using the following equation:

(1) $$\left[5-\mathrm{FU}\right]={\left[5-\mathrm{FU}\right]}_{0\;}\times e^{-k_{ap}^{\prime }\times t}$$

where [5-FU]_0_ and [5-FU] denote the pollutant concentration at *t* = 0 and *t* = *t*, respectively, *k*′*_ap_* is the pseudo-first order kinetic constant, and *t* is the reaction time. The values of *k*′*_ap_* were calculated by non-linear regression.

Total organic carbon (TOC) analysis was performed in the Shimadzu TOC-5000A apparatus (Tokyo, Japan). The TOC content of initial and final samples was studied for selected reactions. Additional photocatalytic experiments were performed to determine the optimum pH solution and photocatalyst stability. The photodegradation of 5-FU was assessed at different pH values, i.e., 3.0, 6.3 (natural), and 9.0, by adding HCl 0.1 M or NaOH 0.1 M, respectively. The photocatalytic degradation pathway of 5-FU was evaluated using methanol (MeOH, 1.0 mM), furfuryl alcohol (FFA, 1.0 mM), and ethylenediaminetetraacetic acid (EDTA, 1.0 mM), as radical, singlet oxygen (^1^O_2_), and hole scavengers, respectively [40,41]. 

## 3. Results

### 3.1. Material Characterization

**Figure 1:**
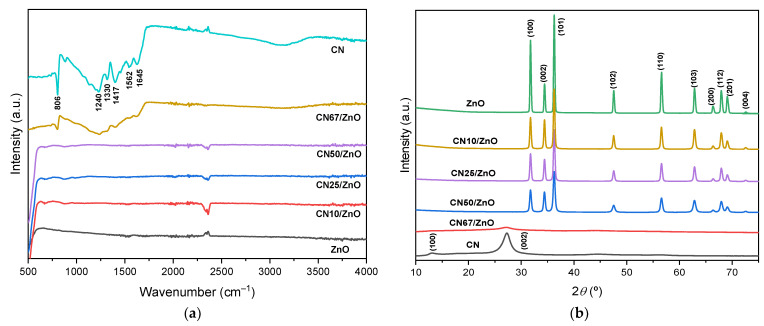
(**a**) FTIR spectra and (**b**) XRD patterns of pure CN, pristine ZnO, and CN/ZnO composites.

The occurrence of both g-C_3_N_4_ and ZnO phases in CN/ZnO composites was studied by ATR-IR. Figure 1a shows the corresponding spectra for pure CN, pristine ZnO, and their composites. Pure CN showed several peaks between 1200–1650 cm^−1^ associated with the typical stretching vibration of g-C_3_N_4_ heterocycles. The bands placed at 806 and 1417 cm^−1^ were assigned to the s-triazine ring modes, while the peaks at around 1330 and 1645 cm^−1^ are attributed to C–N and C=N bonds, respectively. The broad band between 3000–3500 cm^−1^ belongs to deformation and stretching modes of N–H bonds [42,43,44]. As expected, the spectra of ZnO presented a high intensity peak at ~500–550 cm^−1^ due to the stretching vibration of Zn–O bonds [22]. The spectra of CN/ZnO composites show only the band characteristics of CN at high CN content (67 wt%). However, when the presence of Zn–O bonds increases beyond 50 wt%, the predominant bands correspond to the Zn–O vibration.

The crystallinity of the different prepared materials was studied by XRD. The XRD patterns of pure CN, pristine ZnO, and their corresponding composites are depicted in Figure 1b, while the average size of ZnO crystallites (*d*_ZnO_) is collected in Table 1. The CN sample presented the typical diffraction peaks at 2*θ* ~12.7 and 27.2° due to (100) and (200) planes of the structural packing of tris-triazine units and the interlayer stacking of g-C_3_N_4_ aromatic units, respectively [45]. On the other hand, pristine ZnO and CN/ZnO composites with g-C_3_N_4_ contents up to 50 wt% showed major diffraction peaks at 2*θ* values of 31.7, 34.4, 36.2, 47.5, 56.6, 62.8, 66.2, 67.9, 69.0, 72.3, and 77.1°, which were associated with the lattice planes of (100), (002), (101), (102), (110), (103), (200), (112), (201), and (004), respectively, corresponding to the formation of the ZnO wurtzite structure [46]. The intensity of the XRD peaks associated with the wurtzite phase was smaller with the increase of the weight ratio of melamine. During the precursor mixtures, these peaks completely disappear for the composite synthetized with the highest g-C_3_N_4_ content, i.e., for CN67/ZnO (Figure 1b). The disappearance of the XRD peaks which belongs to the ZnO phase was already reported for CN/ZnO composites with g-C_3_N_4_ contents around 50 wt% [26,27,28]. It seems that above this critical percentage the C_3_N_4_ polymeric network hinders the formation of crystalline ZnO particles. Similar results were found for titania/carbon composites. The organic phase certainly avoided the formation of inorganic crystalline structures or even, phase transitions, at metal oxide contents between 15–85% due to the interactions between the different phases along the synthesis procedure [47]. On the other hand, the addition of g-C_3_N_4_ in the ZnO phase had a clear effect on the crystallite size (*d*_ZnO_, Table 1). Therefore, the lower the crystal size, the higher the g-C_3_N_4_ content.

**Figure 2:**
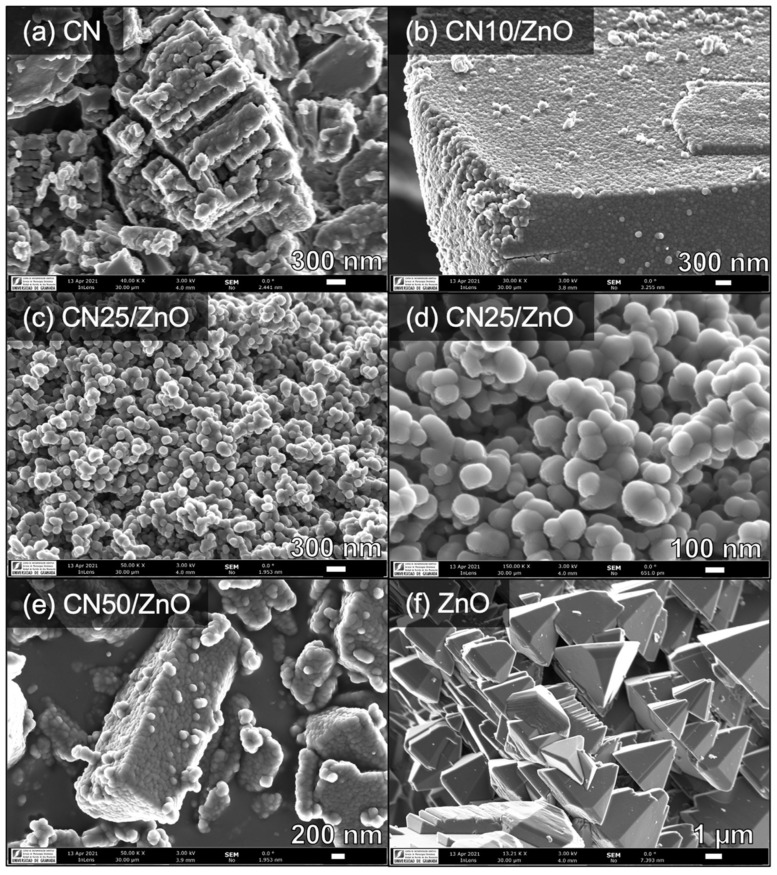
SEM micrographs of (**a**) pure CN, (**b**) CN10/ZnO, (**c**,**d**) CN25/ZnO, (**e**) CN50/ZnO, and (**f**) pristine ZnO.

The morphology of the samples was studied by SEM. Figure 2 shows the corresponding micrographs of pure CN, pristine ZnO, and selected CN/ZnO composites. The structure of bare CN consisted of pillared aggregates with irregular particles (Figure 2a), while particles in the range of 1–1.5 µm with hexagonal pyramid morphology were formed for pristine ZnO (Figure 2f). It is well-known that ZnO can be obtained into a large variety of morphologies, such as nanowires, nanorods, tetrapods, nanobels, nanoflowers, and nanoparticles depending on the synthesis conditions. Under the one-pot synthesis procedure, wurtzite ZnO hexagonal micro-pyramids were majorly obtained. Nevertheless, this morphology strongly changes in the composites, which demonstrated the interactions of both precursors during the calcination treatments. With only 10 wt% of CN in the composite, the morphology was composed by sphere-like particles (Figure 2b), although a trend to form particle agglomerates can be identified as the g-C_3_N_4_ content increased. Of note, the coral-like structure of 3D interconnected ZnO particles for CN25/ZnO, in which well-defined sphere-like particles were clearly identified (Figure 2c,d). With regards to CN50/ZnO, the morphology of the composite resembles again the structure of pure CN (Figure 2e). In general, all of the composites exhibited the g-C_3_N_4_ structure embedded into ZnO. In addition, the crystal size of spherical ZnO particles was smaller with the increase in the wt% of g-C_3_N_4_ content, in agreement with the XRD results. Therefore, the C_3_N_4_ phase hinders the ZnO crystal growth and sintering, in a way that the pyramidal morphology of the ZnO particles was never observed in the composites, independently of the ratio between the phases. This fact is more notorious in CN67/ZnO, where the XRD peaks corresponding to ZnO were not observed (Figure 1), denoting the formation of very small nanocrystals or even, the amorphous character of these particles into the composite.

**Figure 3:**
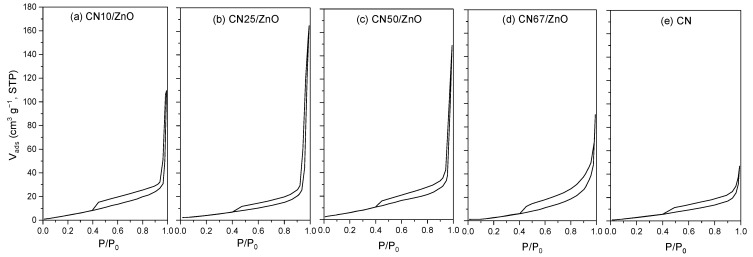
N_2_ adsorption–desorption isotherms of (**a**) CN10/ZnO, (**b**) CN25/ZnO, (**c**) CN50/ZnO, (**d**) CN67/ZnO and (**e**) pure CN.

The textural characterization of the different prepared materials was studied by the physical adsorption of nitrogen at −196 °C. Figure 3 shows the N_2_ adsorption−desorption isotherms of pure CN and CN/ZnO composites. In general, all of the adsorption isotherms can be classified as type-II and type-IV, in concordance with the IUPAC classification, which indicates the presence of macroporosity or low porosity [26], and mesoporosity [48], respectively. Of note, the absence of micropores in all of the samples was negligible, since the adsorbed volume of N_2_ was recorded at low relative pressures. Nevertheless, the presence of large mesopores was notorious in most of the samples, in particular when a 25–50 wt% of g-C_3_N_4_ was used in the composites (Figure 3c,d). This fact was denoted by the high N_2_ volume adsorbed at high relative pressures, as a consequence of the capillary condensation in mesopores. On the other hand, all of the samples showed a small type-H3 hysteresis loop due to aggregates formed by plate-like particles or adsorbents with slit-shaped pores [27,36]. 

All of the synthetized materials exhibited a low BET surface area (*S*_BET_) in the range of 11 and 32 m^2^ g^−1^, due to the absence of micropores. In general, CN/ZnO composites usually show slightly higher *S*_BET_ values than pure CN (e.g., 18 and 29 m^2^ g^−1^ for pure CN and CN50/ZnO, respectively) and pristine ZnO (<5 m^2^ g^−1^). The addition of any amount of g-C_3_N_4_ allowed for the enhancement of the porosity of the composites due to the development of mesoporosity. Therefore, the mesopore volume (*V*_meso_) and total pore volume (*V*_total_) increased as the g-C_3_N_4_ content increased and reached their maximum values of around 25–50 wt% (e.g., *V*_meso_ = 0.19 and 0.09 cm^3^ g^−1^ for CN25/ZnO and CN67/ZnO, respectively). These results are in agreement with the reported studies in literature [27] and those obtained by SEM, in which a more porous structure can be identified for the CN25/ZnO composite.

**Figure 4:**
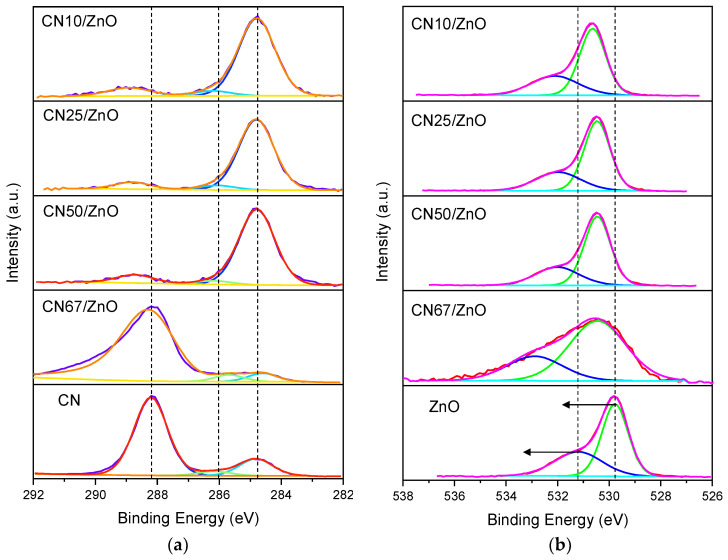
XPS spectra and deconvolution of (**a**) C1s and (**b**) O1s regions of CN/ZnO composites. Pure CN and pristine ZnO are also plotted for comparison.

The surface chemical composition of pure CN, pristine ZnO, and CN/ZnO composites was investigated by XPS (Figure S1a and Table S1). The high resolution C1s spectra of all g-C_3_N_4_-based materials can be deconvoluted in three peaks (Figure 4a). The main peak located at ~284.8 eV was assigned to N–C=N bonds from C-sp^2^ atoms bonded to nitrogen in aromatic rings, while the second peak at ~286.2 eV corresponds to C-sp^3^ atoms from C–(N)_3_ bonds [27]. The last peak placed at ~288.2 eV was attributed to C–N–C bonds [49]. The C1s region was comparable for all of the CN/ZnO composites. However, it is noteworthy to mention the strong change in the C1s profiles (carbon nature) from pure CN to the derivative composites. In bare CN, the large part of C–bonds corresponds to C–N–C structures, which in the presence of the ZnO phase are transformed into N–C=N bonds (Figure 4a), indicating an increase of the aromaticity in the g-C_3_N_4_ phase. This fact is related with a re-ordering of the g-C_3_N_4_ structure, as a consequence of the presence of large amounts of ZnO and the post-treatment in air. In fact, a very small shoulder at ~283 eV seems to be identified for CN/ZnO composites with g-C_3_N_4_ below 50 wt% and corresponding to Zn–C bonds due to oxygen vacancies (O_vac_) formed in the ZnO structure [50,51]. The occurrence of O_vac_ has been reported to have an enhancement in the visible light response of the materials [52]. Concerning the O1s region, the high resolution spectra were deconvoluted in two main contributions (Figure 4b), the first peak located at ~530.5 eV was assigned to O_2_^–^ ions in Zn–O bonds of the ZnO wurtzite structure, while the peak at ~532 eV corresponds to OH groups absorbed onto the ZnO surface [22,27,53]. By comparing the O1s regions of pristine ZnO and CN/ZnO composites, a similar nature of the Zn–O bonds is obtained since the profiles of the spectra are maintained. However, a clear shift is also observed towards higher binding energies (BE), indicating a lower electronic density of the ZnO phase, which is probably associated with the electron transfers between phases or even, a certain reduction of the surface oxygen with the formation of O_vac_ [50]. The proportion of O_2_^–^ groups progressively increased as the g-C_3_N_4_ content increased (Table S1). The N1s spectra were similar for all of the g-C_3_N_4_-based materials and were deconvoluted in three main contributions at ~398.7, ~400.0, and ~401.2 eV attributed to N-sp^2^ (C–N=C bonds) in the triazine rings, tertiary nitrogen atoms from NV(C)_3_ bonds, and amino groups (C–N–H) (Figure S1b) [49]. Regarding the Zn2p region, only a Zn2p_3/2_ peak at ~1021.2 eV associated with a Zn^2+^ oxidation state was found for all of the samples containing ZnO (Figure S1c) [22,27]. The BE difference between Zn2p_3/2_ and Zn2p_1/2_ was always maintained at 23.0 eV. However, a shift towards lower BE is observed for CN/ZnO composites with g-C_3_N_4_ below 50 wt%. This transition occurred by a heterojunction formed between both g-C_3_N_4_ and ZnO phases [50]. Of note, the analysis of the different spectral regions pointed out the stronger similarity of CN67/ZnO with the pure phases, indicating smaller interactions between the phases in this sample. These results point out that the formation of heterojunctions between both semiconductors is favored at g-C_3_N_4_ contents below 50 wt%.

**Figure 5:**
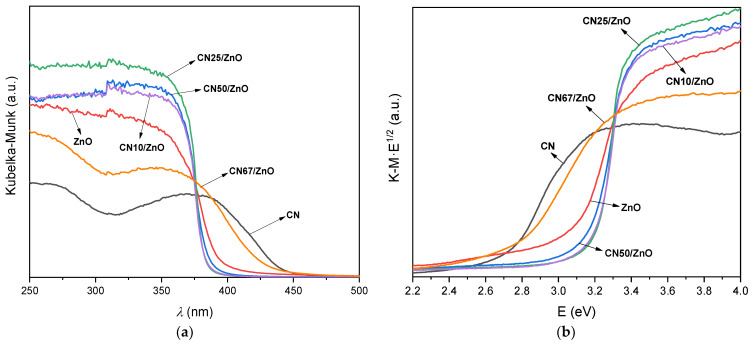
(**a**) UV–Vis spectra and (**b**) Tauc’s plots vs. energy (eV) of pure CN, pristine ZnO, and CN/ZnO composites.

The UV–Vis diffuse reflectance spectra of pure CN and CN/ZnO composites, expressed in terms of Kubelka–Munk absorption units, are depicted in Figure 5a. With regards to all of the composites, a strong intensive absorption band was observed in the UV range with onset at λ < 400 nm, associated with common UV–Vis absorption of pristine ZnO, as previously reported [22]. For the CN material, it also shows a band in the visible range due to π–π * transitions of conjugated ring system [54]. Regarding the ZnO spectrum, an enhanced absorption in the UV range can be observed with the incorporation of g-C_3_N_4_ in the corresponding composites. Figure 5b exhibits the Tauc’s plots vs. the energy (eV). The calculated E_g_ of pure CN and pristine ZnO was 2.65 and 3.0 eV, respectively, which are comparable values to those reported in literature [8]. Nevertheless, when the band gaps of CN/ZnO composites were analyzed, different results were found depending on the g-C_3_N_4_ content. Therefore, the E_g_ value was around 2.70 eV, which is close to the pure CN, at high g-C_3_N_4_ contents (sample CN67/ZnO), while with the increasing ZnO content, the band gap became even higher than for pure ZnO, which is between 3.10–3.15 eV (Table 1). The presented CN and CN67/ZnO developed adsorption at λ > 375 nm, while with the increasing ZnO ratio in the composite, a clear enhanced UV-absorption in the range of 300–400 nm is evident. These results should be due to the interactions between both phases and their transformations during the synthesis procedure, as aforementioned, which are related to the quantum size of active nanoparticles and/or electronic interphase effects. As previously mentioned, the higher the g-C_3_N_4_ content in the composite, the smaller the interactions between the phases.

### 3.2. Photocatalytic Degradation of 5-FU under UV-LED Irradiation

**Figure 6:**
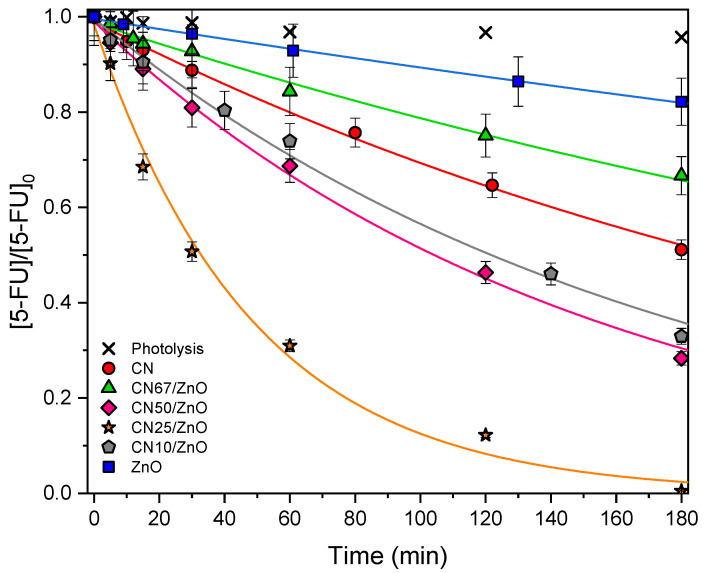
Photocatalytic degradation of 5-FU at natural pH as a function of time for pure CN, pristine ZnO, and CN/ZnO composites. Curves represent the fitting of the pseudo-first order equation to the experimental data.

The photocatalytic performance of pure CN, pristine ZnO, and CN/ZnO composites (with different g-C_3_N_4_ contents) for the 5-FU degradation at natural pH under UV-LED irradiation, is shown in Figure 6. The kinetic rate constant (*k*′*_ap_*), the 5-FU conversion (*X_5-FU_*), coefficient of variation (*k_CV_*), and regression coefficient (r^2^) are summarized in Table 2. The photolysis reaction corroborated the resistance of 5-FU for degradation (X_5-FU_ < 5%) and its light-stability in the absence of any photocatalyst. On the other hand, the adsorption–desorption equilibrium in dark phase experiments was established after 60 min for the prepared catalysts. The adsorption capacity was 3.0%, 4.7%, 1.5%, 14.3%, 9.6%, and 5.5% for CN, ZnO, CN67/ZnO, CN50/ZnO, CN25/ZnO, and CN10/ZnO, respectively. In general, the adsorption capacity of the materials was well-correlated with the porous texture (e.g., SBET), taking into account the comparable neutral surface chemistry of the samples. However, the CN50/ZnO composite presented the highest 5-FU removal by adsorption. This could be due to the presence of very small pores or micropores (*V*_total_–*V*_meso_), which present the largest affinity by the pollutant molecules.

Analyzing the performance of pristine phases, CN is more active than ZnO according to the higher BET surface area and pore volume and a narrower Eg was detected, taking into account the comparable UV absorption under the UV-LED used in the photocatalytic experiments. Nevertheless, the activity in the composites is not directly related with the g-C_3_N_4_ content, which denotes the importance of fitting compositions and physicochemical characteristics along the synthesis procedure. In general, the presence of ZnO in the composites leads to a higher efficiency for the 5-FU degradation under UV-LED irradiation in comparison with both pure CN and pristine ZnO, denoting a clear effect of the synergism between the phases. In fact, the lower photocatalytic activity was obtained for the lowest ZnO content, CN67/ZnO composite (i.e., 33.3 wt% ZnO), increasing the activity as the ZnO content up to 75% (i.e., CN25/ZnO). However, the CN10/ZnO composite (i.e., 90 wt% ZnO) showed a clear effect of over-doping, which leads to a decrease of activity regarding CN25/ZnO. However, it was clearly not only more active than pristine ZnO, but also than pure CN. As indicated, CN25/ZnO presented the best performance among all of the composites, which was due to an adequate combination of composition, morphology, homogeneity, and enhanced porous texture (this sample presented the highest mesopore volume), intermediate crystal size, and strong interaction between phases, which significantly influence on heterojunctions formation inducing changes in separation photogenerated charges and consequently, on the photocatalytic activity [28,55].

The mineralization degree of the different materials was studied for the photodegradation of 5-FU. Therefore, TOC reduction was also determined at the end of the photocatalytic experiments for the different catalysts under UV-LED irradiation (180 min). In general, the level of mineralization exhibited a similar trend to what was observed for the 5-FU conversion, i.e., CN67/ZnO, CN50/ZnO, CN25/ZnO, CN10/ZnO, CN, and ZnO led to TOC reductions of 15%, 31%, 48%, 27%, 18%, and 8%, respectively, under UV-LED irradiation. 

The most active photocatalyst, i.e., CN25/ZnO composite, was selected for the study of different operating parameters and the photocatalytic mechanism.

#### 3.2.1. Influence of pH Value on 5-FU Degradation

**Figure 7:**
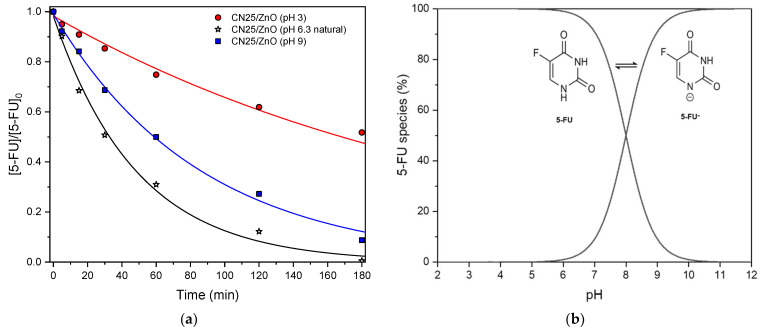
(**a**) Normalized concentration of 5-FU as a function of time pH values of 3.0, 6.3 (natural pH), and 9.0 for CN25/ZnO. (**b**) The 5-FU species distribution diagram as a function of pH.

The solution pH in the photocatalytic experiments affects the surface charge properties of the catalyst, the charge of pollutants, the adsorption onto the photocatalyst surface, the size of aggregate particles formed, as well as the concentration of radical species. Therefore, the effect of initial pH values for the degradation of 5-FU was evaluated in the pH range from 3.0 to 9.0, taking as reference the previously discussed experiments carried out at natural pH (i.e., 6.3), under UV-LED irradiation for the CN25/ZnO photocatalyst (Figure 7a). The respective pseudo-first order kinetic rate constant (*k*′*_ap_*), coefficient of variation (*k_CV_*), and regression coefficient at different pH values can be also found in Table 2. Of note, a slight decrease in the pH value was observed after the photocatalytic experiments (around 0.5). 

Figure 7b shows the species distribution diagram of 5-FU as a function of pH. The diagram exhibits the equilibrium between the neutral (5-FU) and deprotonated form of 5-FU (anionic 5-FU^–^). The neutral form of 5-FU predominates below pH 8.0 and the deprotonated form (5-FU^–^) is mainly observed above pH 8.0 [56]. On the other hand, the pH_PZC_ of the composites is in the range of 7.0–7.4, which indicates that the composite surface is positively or negatively charged for lower or higher pH values, respectively. In this context, the obtained results indicate that the highest photocatalytic activity of the composites is obtained at natural pH values, since the 5-FU removal increased as the media pH value from pH 3.0 to 6.3 (48.3% and 100.0%, respectively). In this situation, the 5-FU molecules remain neutral (uncharged), while the catalyst surface is slightly negatively charged. Therefore, the adsorption is mostly governed by the π–π interactions of the aromatic structures, which were enhanced with the presence of ZnO, in agreement with the XPS results. The lowest activity obtained at pH 3.0 should be related with the agglomeration of the photocatalyst particles and a reduction of the available surface area [57], as well as the presence of chlorine species (from HCl added to adjust the pH value), that can compete with the pollutant molecules for the generated oxidative species [58]. On the contrary, at pH 9.0, both the CN25/ZnO photocatalyst and the 5-FU molecules are negatively charged (pKa = 8.0) and electrostatic repulsions occur, explaining the lower 5-FU conversion obtained at basic pH value. However, a higher 5-FU conversion was obtained at pH 9.0 when compared with pH 3.0. These results can be due to the presence of hydroxyl anions which can lead to the formation of hydroxyl radicals through the reaction with positive holes, resulting in an enhancement of the photocatalytic performance [59,60].

#### 3.2.2. Photocatalytic Degradation Pathway and Reaction Mechanism

**Figure 8:**
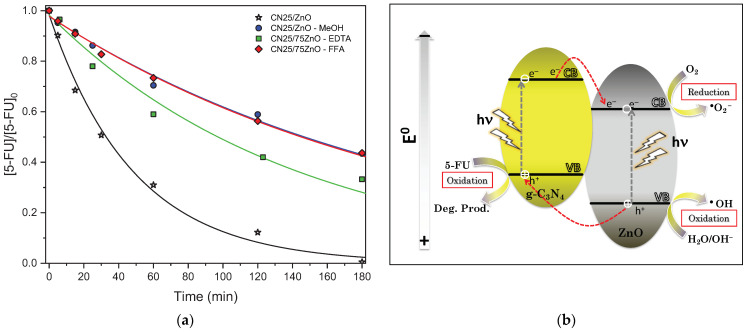
(**a**) Effect of radical/hole/non-radical scavengers (MeOH/EDTA/FFA) on the photocatalytic degradation of 5-FU using the CN25/ZnO composite. (**b**) Scheme of the tentative photocatalytic mechanism with CN/ZnO composites.

MeOH, FFA, and EDTA were used as scavengers for radicals, non-radicals (singlet oxygen), and holes, respectively, in order to study the possible reactive oxygen species (ROS) involved in the photodegradation of 5-FU under UV-LED irradiation (Figure 8a). 

Figure 8a shows that all of the scavengers added to the reaction fulfilled their role and decreased the 5-FU degradation after 180 min of UV-LED irradiation. In particular, this effect was more pronounced in the presence of MeOH and FFA and to a lesser extent by EDTA, the 5-FU photodegradation was around 56.6%, 56.3%, and 66.8%, respectively, compared with 100% of 5-FU degradation in the absence of any scavenger (Table 2). The results suggest that the radicals (mainly hydroxyl radicals) and non-radical species such as, singlet oxygen appear to be the main responsible active species in the degradation of 5-FU, while photogenerated holes play a secondary role in the 5-FU photodegradation [27].

An additional experiment with the CN25/ZnO composite was carried out to study the role of the dissolved oxygen in the generation of ROS involved in the mechanism reaction. In this context, the degradation of 5-FU was studied by changing the pure gas from oxygen to nitrogen, i.e., from reactive and inert atmospheres (Figure S2, Supplementary Materials). The photocatalytic activity with nitrogen purge was almost half compared with the oxygen flow (i.e., 54.9% vs. 100%, respectively), which demonstrated the marked influence of dissolved oxygen in the generation of ROS for the 5-FU degradation under UV-LED irradiation.

Taking into consideration the ROS active in the photocatalytic reactions, the physico-chemical properties of the CN/ZnO composites and based on the related literature, a tentative photocatalytic mechanism is proposed in Figure 8b. The g-C_3_N_4_ is a typical visible light-driven photocatalyst, although a certain UV absorption under near UV-LED irradiation was observed (Figure 5b). As known, the conduction band (CB) potential of pure g-C_3_N_4_ is more negative than the pristine ZnO (i.e., −1.12 V vs. −0.5 V, respectively) [27,32,61]. Therefore, the electrons may be excited to CB of pure g-C_3_N_4_, while holes are generated in the corresponding valance band (VB). Thereafter, the photogenerated electrons are transferred to CB of pristine ZnO, as a consequence of the heterojunction interface produced during the thermal treatment. Of note, the generated holes into g-C_3_N_4_ are not able to directly oxidize ^‾^OH or H_2_O into **^−^**OH, due to the fact that its VB edge potential is less positive compared with the standard redox potential of ^‾^OH/**^−^**OH (2.38 V) or H_2_O/**^−^**OH (1.99 V) [62,63]. However, they can oxidize the 5-FU molecules into degradation products. On the other hand, ZnO can be also excited upon irradiating the composite. In this case, the corresponding holes generated in its VB are able to form **^−^**OH radicals and/or be transferred to the VB of g-C_3_N_4_ [64]. Therefore, a separation of the photogenerated charge carries was achieved, while holes or electrons can react with water or dissolved oxygen to yield hydroxyl or superoxide radicals, respectively, and consequently, degrading the 5-FU molecules as follows:

e_CB_^–^ + O_2_ → O_2_^−^^‾^ (2)

h_VB_^+^ + ^‾^OH → **^−^**OH (3)

O_2_^−^^‾^ + H_2_O → **^−^**O_2_H + ^‾^OH (4)

**^−^**O_2_H + H_2_O → H_2_O_2_ + **^−^**OH (5)

H_2_O_2_ → 2 **^−^**OH (6)

5-FU + **^−^**OH → CO_2_ + H_2_O (7)

5-FU + h_VB_^+^ → Deg. Prod. (8)

#### 3.2.3. Reutilization Tests

**Figure 9:**
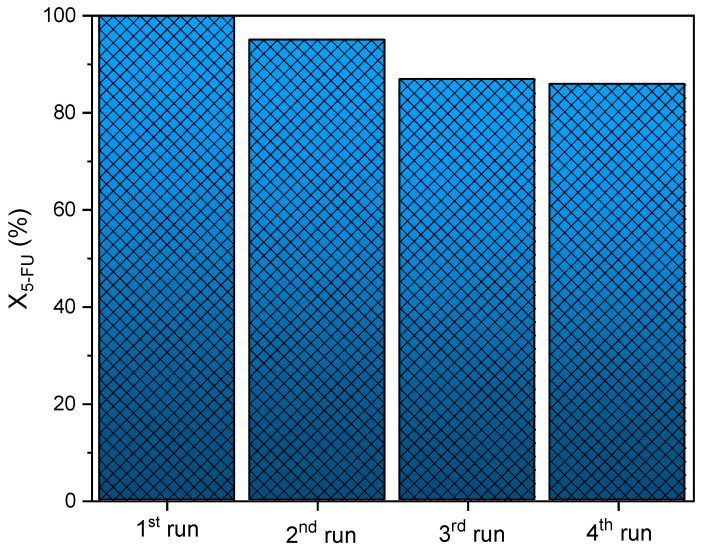
Reusability of CN25/ZnO photocatalyst for the 5-FU degradation during four consecutive cycles.

The photostability of the CN25/ZnO composite was assessed in several consecutive reusability cycles under UV-LED irradiation for 180 min (Figure 9 and Table 2). The experimental procedure was described in Section 2.3. However, after each reaction, the photocatalyst was washed with distilled water and dried at 60 °C overnight. The resulting catalyst was reused in the photocatalytic reactions using a fresh 5-FU solution. The 5-FU conversion values decreased slightly between the first and third run (from 100.0% to 86.9%). However, the photocatalytic activity remained practically constant after the third and fourth cycles (86.9% and 85.8%, respectively). This small deactivation could be related with the chemisorption of 5-FU molecules or reaction intermediates on the stronger active sites of the photocatalyst, remaining after the saturation of the available sites for consecutive reactions. The results showed that the composite can be used to stabilize ZnO in photodegradation processes. Evidently, in this case, there was no photo corrosion, as the photocatalyst exhibited good photostability and performance to remove 5-FU under UV-LED irradiation, even after 12 h of use [26,28,32].

Table 3 summarizes the results obtained in this work with those reported in literature. Some experimental details, such as the procedure synthesis, the precursors, the pollutant studied, the light source employed, and the pollutant removal at a determined time are listed. The results obtained with the best photocatalyst, i.e., CN25/ZnO, are in agreement with the values reported in the literature, although the similarity as well as the difference can be observed.

## 4. Conclusions

The easy one-pot thermal method used to synthetize the CN/ZnO composites favored coral-like structures of spherical particles of ZnO on the g-C_3_N_4_ structure. The morphology of these composites depended markedly on the g-C_3_N_4_ content, in which a homogenous distribution of nanoparticles was achieved preferentially up to 25 wt% of g-C_3_N_4_. The crystallite size of ZnO decreased contrary to the g-C_3_N_4_ content, although the wurtzite structure was identified in most of the composites. The porosity and surface area of the materials were low, although an enhancement of the mesoporosity was always favored with the g-C_3_N_4_ addition. The main chemical functionalities identified in the CN/ZnO composites were mainly formed by N–C=N bonds, as a consequence of a re-ordering of the g-C_3_N_4_ structure due to the presence ZnO and the thermal exfoliation in air. The shift of certain functionalities towards lower BE suggested the occurrence of a heterojunction interface between g-C_3_N_4_ and ZnO phases, with these phase connections as well-stablished for CN/ZnO composites with a g-C_3_N_4_ content below 50 wt%. All of the CN/ZnO composites showed higher light absorption in the UV range in comparison with the reference materials.

All of the synthetized CN/ZnO composites showed photocatalytic activity for the degradation of 5-FU under UV-LED irradiation. The best composite was prepared with 25 wt% of g-C_3_N_4_ (i.e., CN25/ZnO) due to a smaller size and better distribution of ZnO crystallites on the g-C_3_N_4_ structure, an enhanced mesoporosity, and heterojunction interface, which hinders or decreases the electron–hole recombination. The catalytic results obtained at different pH values confirmed that the 5-FU removal was enhanced at natural pH due to the π–π interactions established between the 5-FU molecules and the catalyst surface. The employment of different scavengers allowed for the elucidation of the ROS involved in the photocatalytic mechanism. Therefore, radicals (mainly hydroxyl radicals) and non-radical species (singlet oxygen) seem to be the main responsible active species in the degradation of 5-FU, while photogenerated holes play a secondary role. Furthermore, the occurrence of dissolved oxygen enhanced the photocatalytic activity of the composites due to the generation of ROS. In the tentative photocatalytic mechanism, the photogenerated holes in VB of ZnO under UV-LED are transferred to VB of g-C_3_N_4_, while its photogenerated electrons are transferred to CB of ZnO, as a consequence of the heterojunction interface produced during the thermal treatment of CN/ZnO composites. 

Overall, CN25/ZnO showed high photostability and performance for the degradation of 5-FU during four consecutive reaction cycles.

## Figures and Tables

**Table 1:** Textural, chemical, and optical properties of pure CN, pristine ZnO, and CN/ZnO composites.

Samples	*d*_ZnO_ (nm)	*S*_BET_ (m^2^ g^−1^)	*V*_meso_ (cm^3^ g^−1^)	*V*_total_ (cm^3^ g^−1^)	pH_PZC_	E_g_ (eV)
CN	-	18	0.04	0.07	6.3	2.65
ZnO	32.3	<5	-	-	7.5	3.00
CN10/ZnO	28.1	11	0.09	0.17	7.4	3.10
CN25/ZnO	26.3	21	0.19	0.26	7.2	3.10
CN50/ZnO	21.8	29	0.03	0.23	7.1	3.15
CN67/ZnO	-	32	0.09	0.12	7.0	2.70

*S*_BET_: BET surface area; *d*_ZnO_: Average crystal size; *V*_meso_: Mesopore volume; *V*_total_: Total pore volume; pH_PZC_: pH at the point of zero charge; E_g_: Band gap energy.

**Table 2:** The 5-FU conversion after 180 min (X_5-FU_), pseudo-first order kinetic rate constant (*k*′*_ap_*), coefficient of variation (*k_CV_*), and regression coefficient (r^2^) of 5-FU.

Catalyst	pH	Scavenger	X_5-FU_ (%)	*k*′*_ap_* (10^−3^ min^−1^)	*k_CV_* (%)	r^2^
Photolysis	6.3	none	4.3	-	-	-
CN	6.3	none	48.9	3.6	2.3	0.997
ZnO	6.3	none	17.8	1.1	2.1	0.998
CN10/ZnO	6.3	none	67.1	5.7	4.4	0.993
CN25/ZnO	6.3	none	100.0	20.6	6.7	0.994
CN50/ZnO	6.3	none	71.7	6.5	3.2	0.997
CN67/ZnO	6.3	none	33.3	2.2	3.1	0.995
CN25/ZnO	3.0	none	48.3	4.0	3.5	0.995
CN25/ZnO	9.0	none	91.3	11.6	4.3	0.996
CN25/ZnO	6.3	MeOH	56.6	4.5	5.4	0.98
CN25/ZnO	6.3	EDTA	66.8	7.0	8.8	0.978
CN25/ZnO	6.3	FFA	56.3	4.6	3.7	0.995

**Table 3:** Compilation of recently published works regarding g-C_3_N_4_/ZnO composites for the photodegradation of pollutants in aqueous solution.

Material	Synthesis	C_3_N_4_/ZnO Precursors	Pollutant	Light Source	Removal (%)/Time (Min)	Ref.
C-doped g-C_3_N_4_/ZnO	Sol-gel + calcination	Dicyandiamide zinc nitrate	MB (10 mg L^−1^)	Xe lamp	78.6/120	[26]
Mesoporous g-C_3_N_4_	Impregnation + calcination	Melamine zinc nitrate	MO (10 mg L^−1^)	W lamp	90.8/120	[27]
C_3_N_4_/ZnO	Mechanical milling + calcination	Melamine zinc oxide	MB (0.01 mM)	Fluorescent lamp Xe lamp	~90.0/180 ~85.0/180	[30]
ZnO/g-C_3_N_4_ nanofibers	Calcination + electrospinning	Melamine zinc acetate	MB (0.01 mM)	Xe lamp	91.8/120	[31]
g-C_3_N_4_/ZnO pencil-like	Calcination + hydrothermal + chemical deposition	Melamine zinc acetate	RhB (7.5 mg L^−1^)	Visible (λ > 420 nm)	94.0/120	[32]
ZnO/g-C_3_N_4_	Sol-gel + calcination	Melamine zinc acetate	AML (10 mg L^−1^)	Fluorescent lamps	53.0/400	[28]
g-C_3_N_4_/ZnO nanorods	Calcination + sol-gel + hydrothermal	Melamine zinc acetate	MO (20 mg L^−1^)	Xe lamp	85.7/30	[65]
CN/ZnO	Calcination	Melamine zinc nitrate	5-FU (154 mM)	LED–UV	100.0/180	This work

MB: Methylene blue; MO: Methyl orange; RhB: Rhodamine B; AML: Amiloride; 5-FU: 5-Fluorouracil.

## Data Availability

Not applicable.

## Supplementary Materials

The following supporting information can be downloaded at: https://www.mdpi.com/article/10.3390/nano12030340/s1: Table S1: Percentages of species and corresponding binding energies (in brackets, eV) of pure CN, pristine ZnO, and the different CN/ZnO composites determined by XPS analysis; Figure S1: XPS spectra of pure CN, pristine ZnO, and the CN67/ZnO composite: (a) Survey, (b) N1s region, and (c) Zn2p region; Figure S2: Photocatalytic degradation of 5-FU with different types of purge gas (O2 and N2) for the CN25/ZnO composite.
