# Supplementary material for: One-Pot Thermal Synthesis of g-C3N4/ZnO Composites for the Degradation of 5-Fluoruracil Cytostatic Drug under UV-LED Irradiation
Source: Nanomaterials (Basel). 2022 Jan 21;12(3):340. doi: 10.3390/nano12030340 (PMC8838034; doi:10.3390/nano12030340)
Supplement: Supplementary file 1 [file nanomaterials-12-00340-s001.zip › nanomaterials-1490857-supplementary.pdf]

nanomaterials

MDPI

Supplementary Information

One-Pot Thermal Synthesis of g-C_3N_4/ZnO Composites for the Degradation of 5-Fluoruracil Cytostatic Drug under UV-LED Irradiation

Álvaro Pérez-Molina, Luisa M. Pastrana-Martínez, Lorena T. Pérez-Poyatos, Sergio Morales-Torres*, and Francisco J. Maldonado-Hódar

NanoTech—Nanomaterials and Sustainable Chemical Technologies, Department of Inorganic Chemistry, Faculty of Sciences, University of Granada; Avda. Fuente Nueva, s/n. ES18071 Granada, Spain; alpemo@ugr.es (Á.P.-M.); lpastrana@ugr.es (L.M.P.-M.); lorenaperez@correo.ugr.es (L.T.P.-P.); fjmaldon@ugr.es (F.J.M.-H.)

* Correspondence: semoto@ugr.es; Tel.: +34-958248489

Table S1. Species percentages and corresponding binding energies (in brackets, eV) of pure CN, pristine ZnO and the different CN/ZnO composites determined by XPS analysis.

Sample	C1s (%)			O1s (%)	
	N-C=N	C-$(N)_3$	C-N-C	O_2^-	O–H
CN	12 (284.8)	4 (286.2)	78 (288.2)	60 (532.1)	40 (533.3)
CN67/ZnO	5 (284.3)	4 (286.3)	91 (288.2)	71 (530.5)	29 (532.8)
CN50/ZnO	86 (284.8)	4 (286.2)	10 (288.7)	69 (530.5)	31 (532.0)
CN25/ZnO	85 (284.8)	5 (286.1)	10 (288.8)	68 (530.5)	32 (532.2)
CN10/ZnO	84 (284.8)	5 (286.2)	11 (288.9)	65 (530.6)	35 (532.1)
ZnO	–	–	–	62 (529.8)	38 (531.2)

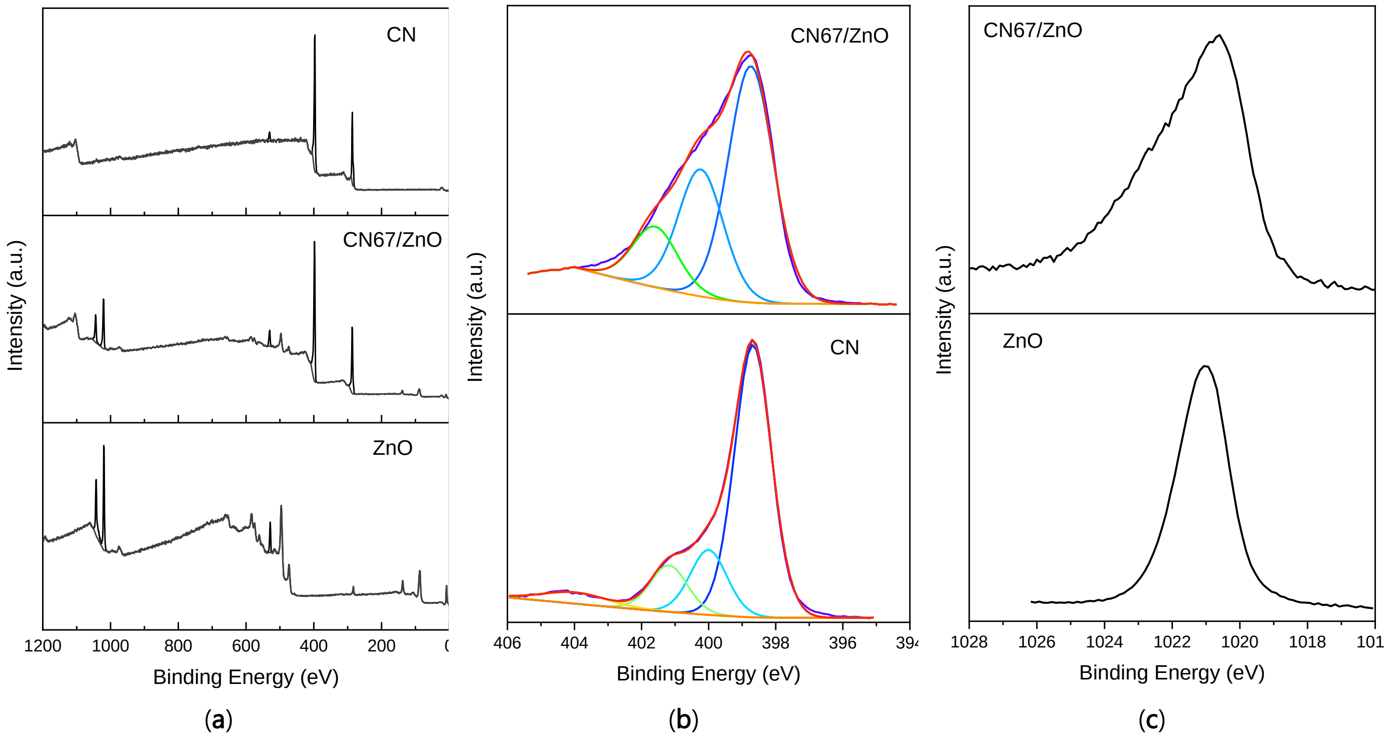

Figure S1. XPS spectra of pure CN, pristine ZnO and the CN67/ZnO composite: (**a**) survey, (**b**) N1s region and (**c**) Zn2p region.

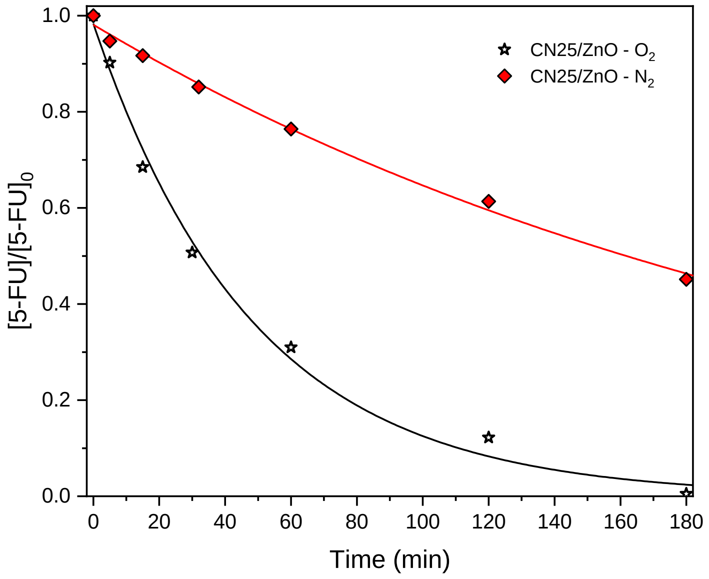

Figure S2. Photocatalytic degradation of 5-FU with different purge gas (O_2 and N_2) for the CN25/ZnO composite.
